# Cardiogenic Shock and the Role of Critical Care Cardiology: Evidence, Models of Care, and Future Directions

**DOI:** 10.1016/j.jscai.2026.104278

**Published:** 2026-03-10

**Authors:** Alexandra N. Schwann, Alexander Ambrosini, Tariq N. Ali, Mark Jacobs, Ann Gage, David Dudzinski, P. Elliott Miller

**Affiliations:** a Section of Cardiovascular Medicine, Department of Internal Medicine, Yale School of Medicine, New Haven, Connecticut; b Centennial Medical Center, Nashville, Tennessee; c Massachusetts General Hospital, Harvard Medical School, Boston, Massachusetts

**Keywords:** cardiac intensive care unit, cardiogenic shock, critical care cardiology, shock teams

## Abstract

The population found in today’s cardiac intensive care unit (CICU) is strikingly different from that found in traditional coronary care units from decades ago, with increasing illness severity and higher prevalence of noncardiac critical illness. Cardiogenic shock (CS) is a heterogeneous syndrome commonly found in modern CICU with a variety of etiologies and presentations; despite advances in noninvasive monitoring and hemodynamic support, as well as efforts to standardize early identification and delivery of therapy, outcomes remain relatively poor. The establishment of critical care cardiology (CCC) as a distinct subspecialty was intended to address the acute needs of the patients in modern CICU, combining cardiovascular medicine expertise with the knowledge and skillset of a critical care intensivist to manage the spectrum of illnesses seen in modern CICU. With this dual expertise, the critical care cardiologist is uniquely positioned to coordinate multidisciplinary care for CS patients—integrating hemodynamic data, knowledge of multiorgan insult and failure driven by shock, procedural skill, and interpersonal influence to foster collaboration and timely decision-making within CS teams. This review aims to provide the history and development of CCC, discuss intensive care unit staffing models, and summarize the evidence supporting CS team utilization. Finally, we propose that continued investment in CCC training, research, and infrastructure is crucial to meet the demands of patients in the modern CICU.

## Introduction—Cardiac intensive care unit transformation

**Figure 1:**
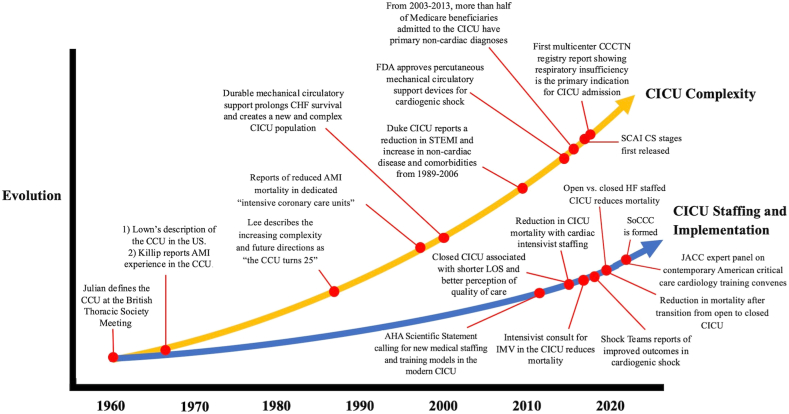
**Evolution and key events for cardiac intensive care unit (CICU) complexity and staffing models.** AHA, American Heart Association; AMI, acute myocardial infarction; CCCTN, Critical Care Cardiology Trials Network; CCU, coronary care unit; CHF, congestive heart failure; CS, cardiogenic shock; FDA, US Food and Drug Administration; HF, heart failure; IMV, invasive mechanical ventilation; JACC, *Journal of the American College of Cardiology*; LOS, length of stay; SCAI, Society for Cardiovascular Angiography & Interventions; SoCCC, Society of Critical Care Cardiology; STEMI, ST-elevation myocardial infarction. Adapted with permission from Quien et al.^29^

The transformation of the coronary care unit (CCU) into the cardiac intensive care unit (CICU) reflects a shift in patient and disease complexity over time. Initially, the CCU primarily managed patients with acute myocardial infarction (AMI) and provided continuous arrhythmia monitoring^1^ (Figure 1). In contrast, contemporary CICU now treat a broader, more critically ill cohort, characterized by increasing age and comorbidity as well as heightened acuity.^2^ Noncardiac diagnoses, including sepsis, respiratory failure, and acute kidney injury, are becoming increasingly common.^3^ Simultaneously, there has been an increasing demand for noncardiac procedures and therapies, such as mechanical ventilation and renal replacement therapy, all of which involve complex pathophysiology and skilled management.^3^, ^4^, ^5^ Cardiogenic shock (CS) accounts for approximately 1 out of every 5 CICU admissions^6^ and remains a particularly formidable challenge, underscoring the need for specialized expertise, multidisciplinary management, and evidence-based strategies. Despite advances in hemodynamic monitoring and invasive hemodynamic support via temporary mechanical circulatory support (tMCS), the majority of randomized control trials (RCT) have failed to identify therapeutic interventions that impact CS outcomes.

In this review, our goal is to highlight the growing role of the critical care cardiologist in the CICU and patients with CS. We will briefly highlight the changing paradigms on staging/phenotyping of CS, discuss evidence for critical care cardiologists and how the field is primed to confront these changes, and future directions for CS management. Importantly, our review highlights evidence and recommendations primarily for a level 1 CICU. We fully recognize that not every institution requires a critical care cardiologist, nor would implementation of critical care cardiology (CCC) staffing be feasible given the currently limited workforce.

## CS in the contemporary era

Cardiogenic shock is a complex, heterogeneous, multifactorial syndrome resulting in insufficient cardiac output, culminating in end-organ hypoperfusion.^7^ Nationally representative data in the United States found that hospitalizations attributed to CS have more than tripled between 2004 (122 per 100,000 hospitalizations) and 2018 (408 per 100,000 hospitalizations).^8^ Despite efforts to improve resuscitation and hemodynamic support in this highly dynamic and unpredictable syndrome, outcomes remain poor, with short-term mortality persisting at approximately 30% to 50%.^8^ Recent strategies to improve outcomes include emphasizing the importance of earlier diagnosis, implementing standardized pathways to expedite and streamline patient care (eg, shock teams), improving risk stratification, and enhancing shock phenotyping.^7^^,^^9^^,^^10^

Given the heterogeneous nature of CS, the Shock Academic Research Consortium convened a multidisciplinary group to differentiate and define CS phenotypes, including acute myocardial infarction–CS and heart failure–CS (HF-CS), de novo HF-CS compared to acute-on-chronic HF-CS, ventricular involvement (right, left, biventricular), and the involvement of noncardiogenic contributors (eg, mixed shock).^11^ Similarly, the Society for Cardiovascular Angiography & Interventions (SCAI) SHOCK stage classification was initially proposed in 2019,^12^ updated in 2022,^13^ and set the stage for researchers and clinicians to “speak the same language,” better define research enrollment, and effectively communicate the time sensitivity and appropriateness of medical and tMCS interventions. Further refinement of the SCAI SHOCK stage classification includes the integration of shock etiology, phenotype, and nonmodifiable risk modifiers, such as age, presence of systemic inflammatory response syndrome, acute kidney injury, and other noncardiac comorbidities.^14^, ^15^, ^16^ The critical care cardiologist plays a central role in identifying the etiology of CS, the initial and repeat staging during early shock management, and “quarterbacking” shock team members and management (Central Illustration).

**Central Illustration:**
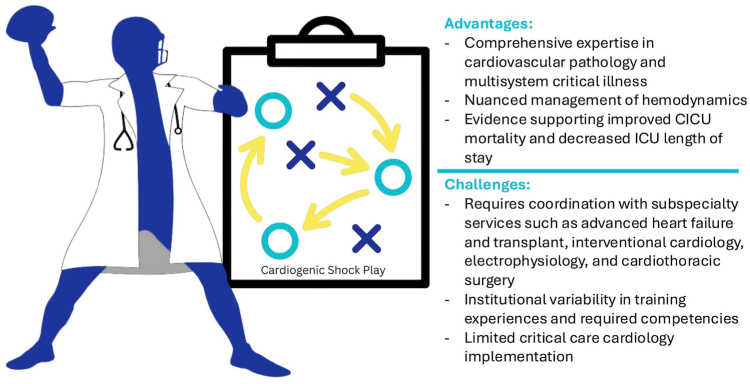
The “quarterback” of the cardiogenic shock team: advantages and challenges of a critical care cardiology lead. CICU, cardiac intensive care unit; ICU, intensive care unit.

It is important to note that the care of the CS patient requires a multidisciplinary team, all of which are crucial to the care of these complicated patients. As CCC evolves as a discipline and profession, in many institutions, the critical care cardiologist, along with analogous colleagues in cardiac anesthesiology, serves as the backbone of the acute cardiovascular team, serving across boundaries of traditional CICU and cardiac surgical intensive care units (ICU). However, it is not uncommon for patients with CS to transfer between units (eg, traditional CICU or cardiac surgical ICU) following deployment of certain tMCS devices, and this can be variable from center to center. This often places other specialties (eg, advanced heart failure/transplant cardiology) as the link, as patients move between ICU.

## The critical care cardiologist

**Figure 2:**
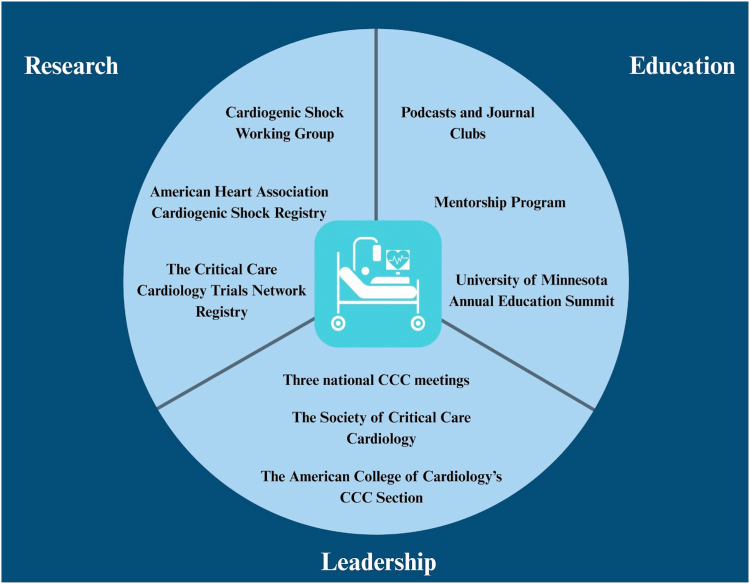
**Successes and achievements of critical care cardiology (CCC).**

The evolution and continued challenges of CS were a huge impetus for the creation of a distinct phenotype of cardiologists dedicated to the care of critically ill patients with cardiovascular disease (CVD),^17^^,^^18^ which has seen substantial growth over the last decade. In 2012, the American Heart Association released a scientific statement emphasizing the importance of dedicated intensivist care in the CICU and described the role of the critical care cardiologist.^19^ A 10-year update, published in 2025 by the American Heart Association, highlighted the progress, successes, and future challenges of the critical care cardiologist.^20^ Among several accomplishments (Figure 2), there are 2 national CCC meetings,^21^^,^^22^ the Critical Care Cardiology Trials Network registry has documented over 20,000 unique admissions to the CICU and expanded internationally,^23^ and the Society of Critical Care Cardiology was created in 2024 to be a home for all clinicians caring for patients with acute CVD.^24^ Furthermore, the American College of Cardiology’s Critical Care Cardiology Section was established in 2022 to support the increasing number of cardiologists interested in pursuing careers focused on the intersection of critical care and cardiovascular medicine, as well as to aid in the development of training programs nationwide.

Although debate remains on the optimal training pathway and clinical competencies, much progress has been made in both domains.^25^ The skillset required in the CICU encompasses both clinical and procedural skills to manage the spectrum of illnesses, including respiratory failure, renal replacement therapy, sepsis and antibiotic stewardship, nutrition, pleural procedures and management, end-of-life care, and much more.^18^ Given these requirements, there is an increasing focus on the validation and standardization of training requirements for CCC. Several concerns remain, including inconsistent fellowship application processes, varying training pathways for certification (3-year CVD fellowship followed by a 12-month critical care medicine [CCM] experience, 2-year CCM fellowship followed by a 3-year CVD fellowship, or integrated programs), and a lack of standardized clinical exposure for trainees because of differences in training environment. Current guidance for trainees wishing to staff level 1 CICU, units equipped to see the highest level of cardiovascular acuity, is to complete individual CVD and CCM fellowships in order to provide dual-board certification in both specialties.^18^

Workforce studies also provide additional insights into the importance of CCC as a subspecialty. A survey was administered to 397 eligible physicians dual-boarded in critical care and cardiovascular medicine, of whom 120 completed the survey, to assess training pathways and career insights. Respondents reported utilizing their critical care training either “regularly” or “all the time” in their current practice. The majority indicated that they felt additional CCM training was necessary in order to effectively practice in a modern CICU setting, and that there was a therapeutic gap in the delivery of care, given the deficiency of cardiologists with adequate CCM training and experience.^26^ These results align with previous data showing that only 14.7% of hospitals had at least 1 critical care cardiologist on staff, with just 8.7% employing them in unit leadership roles.^27^ Further compounding this issue is the limited supply of critical care cardiologists, in part related to the nascency of the field, but also secondary to the scarcity of training availability.^18^

## Staffing models in the CICU

There are numerous staffing models, but 2 general categories of unit staffing models include “open” and “closed” staffing. Open staffing models include multiple attending physicians who admit and manage patients, compared to a closed staffing model, where a dedicated attending, ideally an intensivist, and team care for all patients in the unit. Some have found the term “closed” to have a negative connotation and poorly represent the collaboration of other key stakeholders, including longitudinal relationships with patients’ primary physicians, and prefer the term “high-intensity” staffing.^20^ The evidence to support intensivist or high-intensity staffing, including reductions in mortality, in the ICU is well-documented.^28^ However, data to support staffing models for the CCU and now CICU have been slower to materialize.^29^

In a prospective single-center study of nearly 670 patients at a tertiary care CICU, transition from an open to closed unit was associated with a decrease in ICU length of stay; insight from staff who were surveyed reported improvement in perceptions of communication, collaboration, and education, but noted no significant difference in mortality.^30^ Another single-center study of 4000 patients found that the transition from an open to closed CICU staffing model was associated with a reduction in in-hospital mortality (adjusted odds ratio [OR], 0.69; 95% CI, 0.53-0.90; *P* = .007), but no difference in length of stay or total hospital costs.^31^ In subgroup analysis, patients admitted primarily for cardiac arrest or respiratory failure had a lower in-hospital mortality but primary admissions for CS were not statistically different between staffing models (*P* = .81). In a similar study, staffing of a closed CICU by heart failure physicians was associated with a lower mortality than an open unit with a mixed group of cardiologists, and also in subgroup analysis including patients requiring mechanical circulatory support.^32^ Finally, a South Korean study found that the transition to a CICU fully staffed by critical care cardiologists was associated with a lower mortality, especially for the most critically ill patients (eg, those requiring extracorporeal membrane oxygenation).^33^ To the best of our knowledge, till date, there are no studies evaluating the impact of dual-trained critical care cardiologist staffing in the US. As the number of critical care cardiologists grows and dedicated CICU research grows, future data, such as those from CICU registries, are needed to study the impact of critical care cardiologist staffing on clinical outcomes in the US. Furthermore, as detailed above, the available data include variability in what type of clinician (CCC, advanced heart failure, mix of clinicians) is staffing the unit, highlighting that the current evidence supports the benefits of a single, cohesive team taking care of the entire CICU as opposed to a specific discipline.

## CS teams

Guidelines recommend the establishment of standardized, interdisciplinary shock teams to facilitate early recognition and management of CS, reduce practice variation, and improve outcomes.^7^^,^^34^ The members of the shock teams are variable, but commonly include the CICU attending, an interventional cardiologist, an advanced heart failure and transplant physician, and a cardiothoracic surgeon. In 2019 and 2020, 3 single-center observational studies published their findings after implementing shock teams at their facilities. In each of these studies, the development of a shock team was associated with lower in-hospital mortality.^35^, ^36^, ^37^ Shortly after, a multicenter study from the Critical Care Cardiology Trials Network registry similarly found that CICU with CS teams were associated with lower mortality compared to those without shock teams. They also found important differences in management strategies, including the fact that centers with shock teams were more likely to utilize pulmonary artery catheters and advanced tMCS.^38^ However, each institution and protocol had unique shock team members, processes, and protocols for tMCS selection and escalation.

Although existing guidelines do not specify an optimal CS team leader, the critical care cardiologist is uniquely positioned to serve as the coordinator of the shock team. Analogous to the role of a quarterback (Central Illustration), the critical care cardiologist has the specific knowledge and skillset to direct the multidisciplinary shock team, integrating shock etiology, phenotype, and severity, while also effectively managing other acute comorbid conditions and complications in the CICU (Table 1).^7^^,^^44^, ^45^, ^46^, ^47^, ^48^, ^49^, ^50^, ^51^, ^52^ As modern CICU have evolved, temporal trends have demonstrated increasing noncardiac critical illness—such as sepsis, acute renal failure, and acute respiratory failure—comparable to that seen in general medical ICU.^4^ Management in today’s CICU, therefore, require extensive clinical and procedural expertise in general CCM, as patient outcomes are no longer dictated solely by acute cardiac pathology.^39^

**Table 1:** Complications associated with temporary mechanical circulatory support devices as described in CS randomized controlled trials.

Study, year	Patient Population	Key Comparator	Major Complications reported	Definition/criteria	Incidence (%)
IABP-SHOCK II,^44^ 2012	AMI-CS	IABP vs SOC	1. Life-threatening bleeding 2. Moderate bleeding 3. Vascular complication requiring intervention 4. Ischemic stroke 5. Hemorrhagic stroke	1-2. According to the global use of strategies to open occluded arteries criteria (The GUSTO Investigators^45^)	1. IABP: 10 (3.3%), SOC: 13 (4.4%), *P* = .51 2. IABP: 52 (17.3%), SOC: 49 (16.4%), *P* = .77 3. IABP: 13 (4.3%), SOC: 10 (3.4%), *P* = .53 4. IABP: 2 (0.7%), SOC: 4 (1.3%), *P* = .35 5. IABP: 0, SOC: 1 (0.3%), *P* = .50
Altshock-2,^46^ 2025	HF-CS	IABP vs SOC	1. Bleeding event 2. Vascular complication 3. Limb ischemia 4. Systemic embolism 5. Stroke	1. Bleeding Academic Research Consortium >3 (Mehran et al^47^)	1. IABP: 9 (17%), SOC: 4 (8.3%), *P* = .19 2. IABP: 4 (7.5%), SOC: 0, *P* = .12 3. IABP:2 (3.8%), SOC: 0, *P* = .50 4. IABP: 0, SOC: 1 (2.1%), *P* = .48 5. IABP: 3 (5.7%), SOC: 0, *P* = .24
den Uil et al,^48^ 2019	HF-CS	IABP vs inotropic support	1. Leg ischemia 2. Hemorrhagic stroke 3. Bleeding requiring red blood cell transfusion		1. IABP: 0, inotropes: 0, *P* = .99 2. IABP: 1 (6%), inotropes: 0, *P* = .48 3. IABP: 0, inotropes: 1 (6%), *P* = .99
IMPRESS in Severe Shock,^49^ 2017 (IMPRESS)	AMI-CS	Impella CP vs IABP	1. Device failure 2. Ischemic stroke 3. Major vascular complication 4. Major bleeding 5. Hemolysis requiring device extraction 6. Myocardial reinfarction	1. Any device failure requiring extraction 2. Stroke confirmed by a neurologist on a CT scan 3. Major bleed at access site or limb ischemia prompting extraction, thrombotic femoral artery occlusion, need for vascular surgery intervention 4. Hemoglobin decrease of ≥5 g/dL, bleeding necessitating ≥2 packed cells of product transfusion, or the need for surgical control of bleeding 6. Defined by the Third Universal Definition of MI^50^	1. Impella: 0, IABP: 0 2. Impella: 1 (4%), IABP: 1 (4%) 3. Impella: 1 (4%), IABP: 0 4. Impella: 3 (13%), IABP: 1 (4%) 5. Impella: 2 (8%), IABP: 0 6. Impella: 1 (4%), IABP: 2 (8%)
ISAR-SHOCK,^51^ 2008	AMI-CS	Impella LP2.5 vs IABP	1. Device failure 2. Major bleeding 3. Acute limb ischemia 4. Packed red blood cell transfusion		1. Impella: 0, IABP: 0 2. Impella: 0, IABP: 0 3. Impella: 1 (8.3%), IABP: 0 4. Impella: 2.6 ± 2.7 units, IABP: 1.2 ± 1.9 units, *P* = .18
DanGer Shock,^52^ 2024	AMI-CS	Impella CP vs SOC	1. Composite safety end point^a^ 2. Moderate or severe bleeding 3. Limb ischemia 4. Stroke	2. According to the global use of strategies to open occluded arteries criteria (The GUSTO Investigators^45^)	1. Impella: 43 (24%), SOC: 11 (6.2%) 2. Impella: 39 (21.8%), SOC: 11 (6.2%) 3. Impella 10 (5.6%), SOC: 2 (1.1%) 4. Impella 7 (3.9%), SOC: 4 (2.3%)

AMI, acute myocardial infarction; HF, heart failure; CS, cardiogenic shock; IABP, intra-aortic balloon pump; MI, myocardial infarction; SOC, standard of care.

a Composite safety end point of severe bleeding, limb ischemia, hemolysis, device failure, or worsening aortic regurgitation.

The training of critical care cardiologists across diverse ICU environments provides the necessary foundation to manage varying hemodynamic profiles via noninvasive and invasive tMCS therapies. Responsible providers of CS patients must also be experienced with appropriate tMCS selection and management, as well as prompt recognition and mitigation of device-related complications. Such complications are common, have been associated with increased mortality,^40^^,^^41^ and involve multiple organ systems, with insults categorized broadly as vascular, mechanical, hematologic, infectious, and neurologic.^42^ Given their expertise in acute resuscitation and stabilization across numerous organ systems, critical care cardiologists are uniquely positioned to manage patients supported with tMCS, while addressing the concomitant systemic sequelae of both shock and its therapies. Some critical care cardiologists have also trained in interventional cardiology to develop this dual-minded skillset. Moreover, several dual-fellowship programs now provide trainees with a combined advanced fellowship in CCC and interventional cardiology to hone their clinical and technical expertise.

Effective shock team management demands timely collaboration among a diverse, rapidly changing multidisciplinary provider group. Within this structure, the critical care cardiologist can leverage both authority, ensuring operational efficiency, and influence, fostering an inclusive and cohesive team culture. Although authority derives from organizational hierarchy, influence emerges from trust, relationship building, and the modeling of collaborative behavior. The deliberate balance between authority and influence is crucial in promoting psychological safety and shared situational awareness within a team.

Given the CICU’s highly dynamic environment with frequent rotation of shock team members, success depends not only on structured leadership but also upon teaming—the real-time coordination of multiple disciplines into flexible, goal-oriented groups. Unlike static teams that refine collaboration and trust over time, teaming emphasizes dynamic interactions, adaptive role distribution, and the sharing of expertise to address complex, time-sensitive challenges. Edmondson’s teaming framework underscores the importance of psychological safety, ensuring that all team members can communicate openly and contribute ideas without fear of reprisal.^43^ As the presence of CCC grows in CICU, practitioners should be mindful in modeling effective teaming, demonstrating adaptive leadership, and ensuring the psychological safety of team members, shaping a culture for fast-paced multidisciplinary collaboration in complex clinical scenarios.

## Challenges and future directions

Despite progress, important gaps persist, and outcomes for CS remain poor, with limited high-quality evidence demonstrating therapies that improve outcomes. Investigation continues in risk stratification, the importance of early interventions, and the impact of comprehensive multiorgan failure on CS outcomes. RCT, including novel trial designs, are required to improve the evidence base of CS therapies. Although RCT can be challenging in the acute care environment, there are frameworks and examples from other ICU specialties (eg, medical ICU research on acute respiratory distress syndrome). In addition to CS therapies, the available evidence for staffing models and shock teams is observational and prone to bias. High-quality evidence to define optimal staffing models and protocols is required, and critical care cardiologists should be leaders of these investigations. Clinicians must continue to collaborate with industry as technology advances, with the goal of improving current tMCS devices and decreasing the risk of device-related complications.

Obstacles in CCC training persist because of the lack of fellowship training opportunities and heterogeneity in training pathways, with limited standardization across institutions and unclear required clinical competencies. With the ongoing evolution of the CICU, hybrid training pathways that combine CCC with complementary cardiology subspecialties, including advanced heart failure and interventional cardiology, have emerged to meet the individualized career goals of interested trainees. Ongoing evaluation of optimal pathways and optimal staffing of contemporary cardiac critical care units is required, as well as considering how to best structure clinical footprint and career pathways. Broader societal and professional impacts must also be considered, specifically diversity and inclusion, as training time is extended for combined subspecialty training, and also impacts the unmet need for heart failure cardiologists.

Further work is necessary to create standardized frameworks and appropriate quality metrics for shock care. Variation in institutional resources and personnel requires individualized pathways for timely shock team assembly, decision-making, and execution of stabilizing therapies. These important issues require the dedication of a physician who is trained in and will prioritize their successful implementation. To truly be successful, the CS team must be embedded in the institutional culture and be supported by department and hospital leadership.

Addressing these gaps will require investment in training, recruitment, system redesigns, and robust collaborative multicenter research efforts. Taken together, advancing CCC represents not only an opportunity to improve outcomes in CS but also a fundamental improvement in the delivery of care in the modern CICU.

## Declaration of competing interest

The authors declared no potential conflicts of interest with respect to the research, authorship, and/or publication of this article.

## References

[1] Julian D.G. (1961). Treatment of cardiac arrest in acute myocardial ischaemia and infarction. Lancet.

[2] Miller P.E., Thomas A., Breen T.J. (2021). Prevalence of noncardiac multimorbidity in patients admitted to two cardiac intensive care units and their association with mortality. Am J Med.

[3] Sinha S.S., Sjoding M.W., Sukul D. (2017). Changes in primary noncardiac diagnoses over time among elderly cardiac intensive care unit patients in the United States. Circ Cardiovasc Qual Outcomes.

[4] Katz J.N., Shah B.R., Volz E.M. (2010). Evolution of the coronary care unit: clinical characteristics and temporal trends in healthcare delivery and outcomes. Crit Care Med.

[5] Bohula E.A., Katz J.N., van Diepen S. (2019). Demographics, care patterns, and outcomes of patients admitted to cardiac intensive care units: the Critical Care Cardiology Trials Network prospective North American multicenter registry of cardiac critical illness. JAMA Cardiol.

[6] Berg D.D., Bohula E.A., van Diepen S. (2019). Epidemiology of shock in contemporary cardiac intensive care units. Circ Cardiovasc Qual Outcomes.

[7] Sinha S.S., Morrow D.A., Kapur N.K., Kataria R., Roswell R.O. (2025). 2025 Concise Clinical Guidance: an ACC expert consensus statement on the evaluation and management of cardiogenic shock: a report of the American College of Cardiology Solution Set Oversight Committee. J Am Coll Cardiol.

[8] Osman M., Syed M., Patibandla S. (2021). Fifteen-year trends in incidence of cardiogenic shock hospitalization and in-hospital mortality in the United States. J Am Heart Assoc.

[9] Miller P.E., Huber K., Bohula E.A. (2023). Research priorities in critical care cardiology: *JACC* Expert Panel. J Am Coll Cardiol.

[10] Skove S., Berg D.D., Bohula E.A. (2025). Early evolution of SCAI shock stage and in-hospital mortality in the cardiovascular intensive care unit population: from the Critical Care Cardiology Trials Network (CCCTN). Circ Heart Fail.

[11] Waksman R., Pahuja M., van Diepen S. (2023). Standardized definitions for cardiogenic shock research and mechanical circulatory support devices: scientific expert panel from the Shock Academic Research Consortium (SHARC). Circulation.

[12] Baran D.A., Grines C.L., Bailey S. (2019). SCAI clinical expert consensus statement on the classification of cardiogenic shock: this document was endorsed by the American College of Cardiology (ACC), the American Heart Association (AHA), the Society of Critical Care Medicine (SCCM), and the Society of Thoracic Surgeons (STS) in April 2019. Catheter Cardiovasc Interv.

[13] Naidu S.S., Baran D.A., Jentzer J.C. (2022). SCAI SHOCK stage classification expert consensus update: a review and incorporation of validation studies. J Soc Cardiovasc Angiogr Interv.

[14] Padkins M., Breen T., Van Diepen S., Barsness G., Kashani K., Jentzer J.C. (2021). Incidence and outcomes of acute kidney injury stratified by cardiogenic shock severity. Catheter Cardiovasc Interv.

[15] Jentzer J.C., Lawler P.R., van Diepen S. (2020). Systemic inflammatory response syndrome is associated with increased mortality across the spectrum of shock severity in cardiac intensive care patients. Circ Cardiovasc Qual Outcomes.

[16] Padkins M., Breen T., Anavekar N. (2020). Age and shock severity predict mortality in cardiac intensive care unit patients with and without heart failure. ESC Heart Fail.

[17] Katz J.N., Turer A.T., Becker R.C. (2007). Cardiology and the critical care crisis: a perspective. J Am Coll Cardiol.

[18] Elliott A.M., Bartos J.A., Barnett C.F. (2024). Contemporary training in American critical care cardiology: Minnesota Critical Care Cardiology Education Summit: *JACC* Scientific Expert Panel. J Am Coll Cardiol.

[19] Morrow D.A., Fang J.C., Fintel D.J. (2012). Evolution of critical care cardiology: transformation of the cardiovascular intensive care unit and the emerging need for new medical staffing and training models: a scientific statement from the American Heart Association. Circulation.

[20] Sinha S.S., Geller B.J., Katz J.N. (2025). Evolution of critical care cardiology: an update on structure, care delivery, training, and research paradigms: a scientific statement from the American Heart Association. Circulation.

[21] (2024). NYU Langone Critical Care Cardiology Symposium; October 25-26.

[22] (2025). University of Minnesota Critical Care Cardiology Education Summit.

[23] Metkus T.S., Baird-Zars V.M., Alfonso C.E. (2022). Critical Care Cardiology Trials Network (CCCTN): a cohort profile. Eur Heart J Qual Care Clin Outcomes.

[24] Senman B., Dudzinski D.M., Gage A., Miller P.E., Katz J.N. (2024). The why, the who, and the how: launching the Society of Critical Care Cardiology. J Shock Hemodyn.

[25] Miller P.E., Kenigsberg B.B., Wiley B.M. (2019). Cardiac critical care: training pathways and transition to early career. J Am Coll Cardiol.

[26] Brusca S.B., Barnett C., Barnhart B.J. (2019). Role of critical care medicine training in the cardiovascular intensive care unit: survey responses from dual certified critical care cardiologists. J Am Heart Assoc.

[27] van Diepen S., Fordyce C.B., Wegermann Z.K. (2017). Organizational structure, staffing, resources, and educational initiatives in cardiac intensive care units in the United States: an American Heart Association Acute Cardiac Care Committee and American College of Cardiology Critical Cardiology Working Group cross-sectional survey. Circ Cardiovasc Qual Outcomes.

[28] Pronovost P., Needham D., Berenholtz S. (2006). An intervention to decrease catheter-related bloodstream infections in the ICU. N Engl J Med.

[29] Quien M., Thomas A., Ludmir J., Miller P.E. (2022). Staffing models in the cardiac intensive care unit. Curr Opin Crit Care.

[30] Katz J.N., Lishmanov A., van Diepen S. (2017). Length of stay, mortality, cost, and perceptions of care associated with transition from an open to closed staffing model in the cardiac intensive care unit. Crit Pathw Cardiol.

[31] Miller P.E., Chouairi F., Thomas A. (2021). Transition from an open to closed staffing model in the cardiac intensive care unit improves clinical outcomes. J Am Heart Assoc.

[32] Sims D.B., Kim Y., Kalininskiy A. (2021). Full-time cardiac intensive care unit staffing by heart failure specialists and its association with mortality rates. J Card Fail.

[33] Na S.J., Chung C.R., Jeon K. (2016). Association between presence of a cardiac intensivist and mortality in an adult cardiac care unit. J Am Coll Cardiol.

[34] Blumer V., Hanff T.C., Gage A., Schrage B., Kanwar M.K. (2025). Cardiogenic shock teams: past, present, and future directions. Circ Heart Fail.

[35] Taleb I., Koliopoulou A.G., Tandar A. (2019). Shock team approach in refractory cardiogenic shock requiring short-term mechanical circulatory support: a proof of concept. Circulation.

[36] Tehrani B.N., Truesdell A.G., Sherwood M.W. (2019). Standardized team-based care for cardiogenic shock. J Am Coll Cardiol.

[37] Lee F., Hutson J.H., Boodhwani M. (2020). Multidisciplinary code shock team in cardiogenic shock: a Canadian centre experience. CJC Open.

[38] Papolos A.I., Kenigsberg B.B., Berg D.D. (2021). Management and outcomes of cardiogenic shock in cardiac ICUs with versus without chock teams. J Am Coll Cardiol.

[39] Berg D.D., Singal S., Palazzolo M. (2024). Modes of death in patients with cardiogenic shock in the cardiac intensive care unit: a report from the Critical Care Cardiology Trials Network. J Card Fail.

[40] Ott S., Germinario L., Müller-Wirtz L.M. (2025). Impact of complications on survival outcomes in different temporary mechanical circulatory support techniques: a large retrospective cohort study of cardiac surgical and nonsurgical patients. J Heart Lung Transplant.

[41] Kapur N.K., Whitehead E.H., Thayer K.L., Pahuja M. (2020). The science of safety: complications associated with the use of mechanical circulatory support in cardiogenic shock and best practices to maximize safety. F1000Res.

[42] Geller B.J., Sinha S.S., Kapur N.K. (2022). Escalating and de-escalating temporary mechanical circulatory support in cardiogenic shock: a scientific statement from the American Heart Association. Circulation.

[43] Edmondson A.C. (2012).

[44] Thiele H., Zeymer U., Neumann F.J. (2012). Intraaortic balloon support for myocardial infarction with cardiogenic shock. N Engl J Med.

[45] The GUSTO Investigators (1993). An international randomized trial comparing four thrombolytic strategies for acute myocardial infarction. N Engl J Med.

[46] Morici N., Sacco A., Frea S. (2025). Early intra-aortic balloon support for heart failure-related cardiogenic shock: a randomized clinical trial. J Am Coll Cardiol.

[47] Mehran R., Rao S.V., Bhatt D.L. (2011). Standardized bleeding definitions for cardiovascular clinical trials: a consensus report from the Bleeding Academic Research Consortium. Circulation.

[48] den Uil C.A., Van Mieghem N.M., Bastos M.B. (2019). Primary intra-aortic balloon support versus inotropes for decompensated heart failure and low output: a randomised trial. EuroIntervention.

[49] Ouweneel D.M., Eriksen E., Sjauw K.D. (2017). Percutaneous mechanical circulatory support versus intra-aortic balloon pump in cardiogenic shock after acute myocardial infarction. J Am Coll Cardiol.

[50] Thygesen K., Alpert J.S., Jaffe A.S. (2018). Fourth universal definition of myocardial infarction (2018). Circulation.

[51] Seyfarth M., Sibbing D., Bauer I. (2008). A randomized clinical trial to evaluate the safety and efficacy of a percutaneous left ventricular assist device versus intra-aortic balloon pumping for treatment of cardiogenic shock caused by myocardial infarction. J Am Coll Cardiol.

[52] Møller J.E., Engstrøm T., Jensen L.O. (2024). Microaxial flow pump or standard care in infarct-related cardiogenic shock. N Engl J Med.

